# Development of a PVDF Sensor Array for Measurement of the Dynamic Pressure Field of the Blade Tip in an Axial Flow Compressor

**DOI:** 10.3390/s19061404

**Published:** 2019-03-21

**Authors:** Jiqing Cong, Jianping Jing, Changmin Chen, Zezeng Dai

**Affiliations:** 1 The State Key Laboratory of Mechanical System and Vibration, Shanghai Jiao Tong University, Shanghai 200240, China; sjtu.cjq@sjtu.edu.cn (J.C.); fly__freely@sjtu.edu.cn (C.C.); daizezeng@sjtu.edu.cn (Z.D.); 2 Gas Turbine Research Institute, Shanghai Jiao Tong University, Shanghai 200240, China; 3 Fundamental Science on Vibration, Shock & Noise Laboratory, Shanghai 200240, China

**Keywords:** tip clearance flow, PVDF sensor array, dynamic calibration, dynamic pressure filed measurement

## Abstract

Tip clearance flow in axial flow compressor is unavoidable and responsible for pressure losses and noise generation and influences the stability of the compressor. However, necessary flow measurement in the blade tip region is a great challenge due to the small gap width as well as the structure limitation. In this paper, a polyvinylidene fluoride (PVDF) piezoelectric-film sensor array is developed to capture the dynamic pressure field over the blade tip in an axial flow compressor. The PVDF sensor array with 40 evenly distributed sensing points is fabricated directly on a 30 μm thick aluminum-metalized polarized PVDF film through photolithography. Dynamic calibration of the sensor is accomplished using acoustic source as excitation and a microphone as a reference. The test pressure range is up to 3.5 kPa and the sampling frequency is 20 kHz. The sensor presents a high signal-to-noise ratio and good consistency with the reference microphone. Sensitivity, frequency response, linearity, hysteresis, repeatability as well as the influence of temperature are also investigated through the calibration apparatus. The calibration gives credence to the relevance and reliability of this sensor for the application in dynamic pressure field measurement. The sensor is then applied to an actual measurement in a compressor. The output of the PVDF sensor array is also compared with the results of common pressure transducers, and the features of the dynamic pressure filed are discussed. The results indicate that the PVDF sensor array is capable of the dynamic pressure field measurement over the blade tip, and superior to the conventional approaches in installation, spatial resolution, frequency response, and cost. These advantages indicate its potential broad application in pressure measurement, especially for the complex spatial surface or thin-walled structure, such as the blade surface and the thin casing wall of the compressor.

## 1. Introduction

Tip clearance flow in axial flow compressor is an unavoidable flow induced by the relative motion of the blade tip and the casing. Previous studies had shown that it is responsible for pressure losses [1], noise generation [2], endwall blockage [3], and influences the aerodynamic stability of the compressor [4]. Recent studies also indicate that the tip clearance region is the origin of compressor stall [5] and closely related to the occurrence of blade non-synchronous vibration (NSV) [6,7,8,9]. Therefore, tip clearance flow investigation has been a hot issue in recent years, which is crucial for improving the performance of the compressor and expanding its operating range.

However, it is a great challenge to measure the flow field directly. The geometry size of the blade tip clearance is very small (usually about 0.5–5% of the blade span), which limits the size of the sensor. Moreover, there are a number of casing attachments outside the casing, which brings obstacles to sensor installation and signal output. Hence, a limited number of sensors can be installed. Nowadays, a set of high-response pressure transducers, which are arranged in a line [10,11,12,13] or an array [14,15] on the casing wall over the rotor blade tip, are widely used in tip clearance flow measurement. However, most of these pressure transducers are solid structures, and they usually flush-mounted on the surface. Hence it is inevitable to damage the compressor structure and thus influence the structural strength of the casing. This installation further limits the number of transducers that can be installed. Moreover, the flow field may be also influenced by the measuring holes. Furthermore, the use of numerous transducers is not only costly but also troublesome. Hence, it is necessary to develop a pressure sensor for dynamic pressure field measurement, which needs to fulfill the following requirements: high spatial and temporal resolution, easy install, a wide pressure range, small response time, low cost and have little effect on the compressor structure as well as the flow field. Pressure sensors which are available on the market cannot completely fulfill the above requirements, so in this paper, a pressure sensor based on the polyvinylidene fluoride (PVDF) piezoelectric-film is developed to solve the problem.

The PVDF piezoelectric-film sensor (shortly for PVDF sensor) is fabricated based on its piezoelectric effect, which is the ability of certain materials to generate an electric charge across its boundaries in response to applied mechanical stress [16]. In view of its biocompatibility, flexible, low cost, wide-frequency response, light weight and other outstanding properties [17], it has been used for a variety of physical measurements [18,19,20]. However, most applications are qualitative due to the lack of effective calibration methods. Therefore, the calibration of the PDVF sensor is one of the most important issues for the dynamic pressure field measurement. People usually take the piezoelectric constant *d*_33_ of the PVDF material as the sensor sensitivity, but they are not identical due to the presence of electrodes, protective coat and even the influence of fabrication process. Previous study has shown that they may differ by as much as half [21]. The PVDF sensor is a piezoelectric sensor that can only measure dynamic pressure signals, this is owing to the fact that the charge generated on the electrodes by straining the PDVF film is discharging slowly. Hence only dynamic characteristics need to be considered in the calibration. For transient response calibration, dropping hammer [22], SHPB (split Hopkinson pressure bar) [23] and shock tube [24] are usually adopted. For steady-response calibration, there is almost no literature that discusses this aspect. Sullivan et al. [25,26] and Lee et al. [27] used the sound pressure as the excitation and the frequency response function (FRF) between the sensor and the microphone for quantification. However, the frequency range and pressure range of their calibrations cannot fulfill the requirement in this paper. Moreover, the influence of temperature was out of consideration. Inspired by them, a new calibration method is proposed in this paper.

In this paper, an effective, easy installation, high-resolution, and low-cost PVDF sensor array is developed to capture the dynamic pressure field over the blade tip in an axial flow compressor. The fabrication and dynamic calibration of the PVDF sensor have been illustrated first, which give credence to the relevance and reliability of this sensor for application in dynamic pressure field measurement. Sensitivity, frequency response, linearity, hysteresis, repeatability as well as the influence of temperature are investigated through the calibration apparatus. The PVDF sensor array is then applied in an actual compressor. Single sensing point result is compared with the high-response pressure transducers in the time and frequency domain to verify the effectiveness of the sensor. The dynamic pressure field is measured by the sensor, and the features of the dynamic pressure filed are discussed.

## 2. Sensor Fabrication

The sensor material, PVDF film, is produced by Jinzhou Kexin Electronic Material Co., Ltd. (Jinzhou, Liaoning, China). The basic parameters of the material are shown in Table 1 [28]. It can be seen from Table 1 that sensors made of PVDF material have the capability to measure pressure up to 20 GPa and frequency up to 10^8^ Hz. Actually, in our test, the internal dynamic pressure is below 3.5 kPa, and the main frequency components of interest are in the range of 10 kHz. Hence, the actual required range of the properties is calibrated considering the necessity and cost. For the test compressor, the maximum internal temperature of the first stage may reach 50–60°C, which is below the maximum operating temperature of the PVDF material. However, as the PVDF material is sensitive to temperature, the influence of temperature must be considered in the calibration.

**Figure 1:**
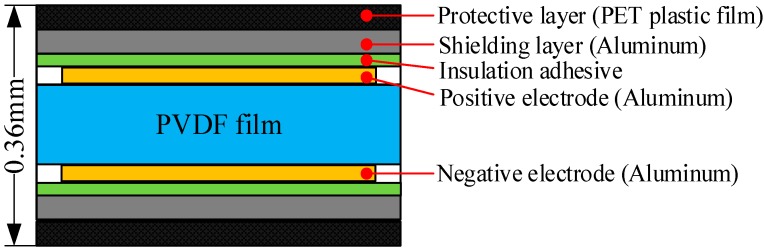
Cross-sectional view of the PVDF sensor (single sensing point).

**Figure 2:**
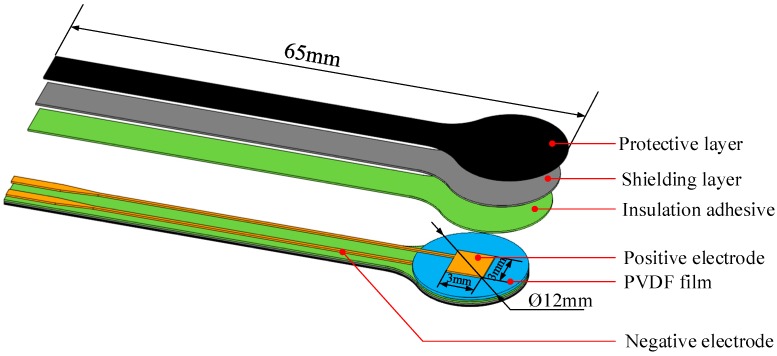
Expanded view of the PVDF sensor (single sensing point).

Since the material, basic structure and manufacturing technique of single point are identical with multi-point, PVDF sensor with a single sensing point is presented in the sensor fabrication and calibration section for illustration purposes. The sensor consists of a 30-µm-thick PVDF film with aluminum electrodes, two shielding layers, and two protective layers. Cross-sectional view and expanded view of the sensor are shown in Figure 1 and Figure 2, respectively. The sensitive part of the sensor is the area where the positive and negative electrodes overlap, that is, the 3 mm × 3 mm square in Figure 2. The final thickness of the PVDF sensor is 0.36 mm, which gives minimal disturbance to the flow field. The top layer and bottom layer are protective layers or insulating layers made of PET plastic film and used to protect the main body of the sensor from wear or erosion. The shielding layers are next to the protective layers and made of aluminum. They are mainly used to shield the sensor against the background noise that comes from the test site and the electromagnetic interference. The PVDF sensor is susceptible to electro-magnetic interference due to the capacitive nature of the sensor. The extraction electrodes are made of aluminum and fall into two parts, positive electrode and negative electrode, and the PVDF film is sandwiched between them. The extraction electrodes and the shielding layer are bonded together by insulation adhesive.

**Figure 3:**
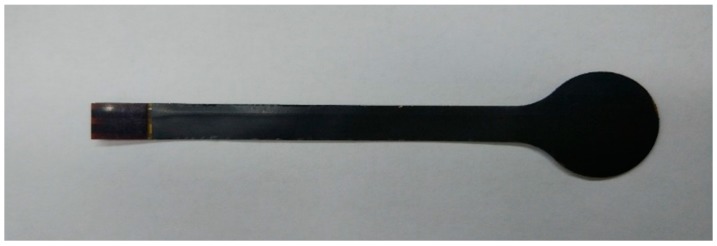
Photograph of the PVDF sensor (single sensing point).

The electrodes on the PVDF film, which represents a single sensing point, are fabricated by photolithography with the pattern of positive and negative electrodes. Since the resolution of the lithography equipment (SSB260/20T, Shanghai Micro Electronics Equipment Co., Ltd., Shanghai, China) is 1.5 μm, the size of each single sensing point, as well as the distance between them, can be very small. After encapsulation, the sensor is cut into the designed shape by laser. The photograph of the sensor is shown in Figure 3.

Since the PVDF sensor has a soft texture and a low thickness (0.36 mm), it has a potentially broad application in pressure measurement, especially for the complex spatial surface or thin-walled structure, such as the blade surface and the thin casing wall of the compressor, in which the common pressure transducers are difficult to install and measure. Moreover, it has some advantages over the common pressure transducers in cost, installation, spatial resolution and frequency response. For better illustration, a detailed and quantitative comparison between them is shown in Table 2. Kulite XCQ-062 and Endevco 8507C-15, two miniature pressure transducers usually used for casing wall pressure measurement, are selected for the comparison. Two sheet type pressure transducers used for blade surface pressure measurement, Kulite LQ-062 and Endevco 8515C-50, are also included in the comparison.

Cost: The cost of the PVDF sensor mainly depends on the PVDF material and the photolithography mask. After mass production, the cost of a multi-point PVDF sensor array is about RMB 500, which is much cheaper than the Kulite or Endevco transducers. The cost will be further cheaper than the common pressure transducers with the apportion to each sensing point. Moreover, the increase of the sensing points does not have much influence on the cost of the PVDF sensor.

Installation: Most of the common pressure transducers are solid structures, and they are usually flush-mounted on the surface. Hence, it is inevitable to damage the compressor structure and thus influence the structural strength of the casing. This installation further limits the number of sensors that can be installed. Moreover, the flow field may be also influenced by the measuring holes. On the contrary, the PVDF sensor has a soft texture and a thickness of 0.36 mm. When attached to a surface, especially the curved surface in this experiment, the flexibility, and smoothness of the sensor give minimal disturbance to the surface and the flow field. Even compared with the sheet type pressure transducers used for blade surface pressure measurement, the PVDF sensor is less than half of their thickness. Therefore, it has a potentially broad application in pressure measurement, especially for the complex spatial surface or thin-walled structure, in which the common pressure transducers are difficult to install and measure.

Spatial Resolution: Each positive electrode of the PVDF sensor array, which represents a single sensing point, is fabricated by photolithography. The resolution of the lithography equipment is 1.5 μm. Hence from a fabrication point of view, single sensing point, as well as the distance between them, can be small enough, even smaller than 1 mm. Therefore, the PVDF sensor has a potentially higher spatial resolution than the common pressure transducers.

The output signal of a single sensing point comes from the charges generated by the resultant force in the whole sensing point area. In other words, under the same pressure, the larger the area, the greater the resultant force, and the more the output charges. Therefore, the sensing point must be large enough to obtain an ideal output signal with good signal-to-noise ratio. In order to meet the needs of the measurement, the area of one sensing point should be above 4 mm^2^ to get a 20 dB of the signal-to-noise ratio in terms of the present calibration results. Assuming that the sensing point is circular, the diameter should be greater than 2.3 mm.

Frequency Response: In this paper, the frequency is tested up to 10 kHz, which is enough for the pressure measurement in the compressor. But according to the basic parameters of the PVDF material, its frequency response ranges from 10^−2^ to 10^8^ Hz, which may be applied for higher frequency measurement.

Pressure Range: Although the sensitivity calibration is performed in the range of 0–3.5 kPa in this study, the pressure range of the PVDF material can be up to 20 Gpa, as the manufacturer offered. Wen et al. [29] also studied the electrical response of PVDF film under shock loading experimentally, and the result indicated that the released charge increases linearly with the shock pressure in the range of 0–20 GPa. Hence, the PVDF sensor has the potential to be used in a higher-pressure measurement.

## 3. Sensor Calibration

### 3.1. Calibration Apparatus and Method

**Figure 4:**
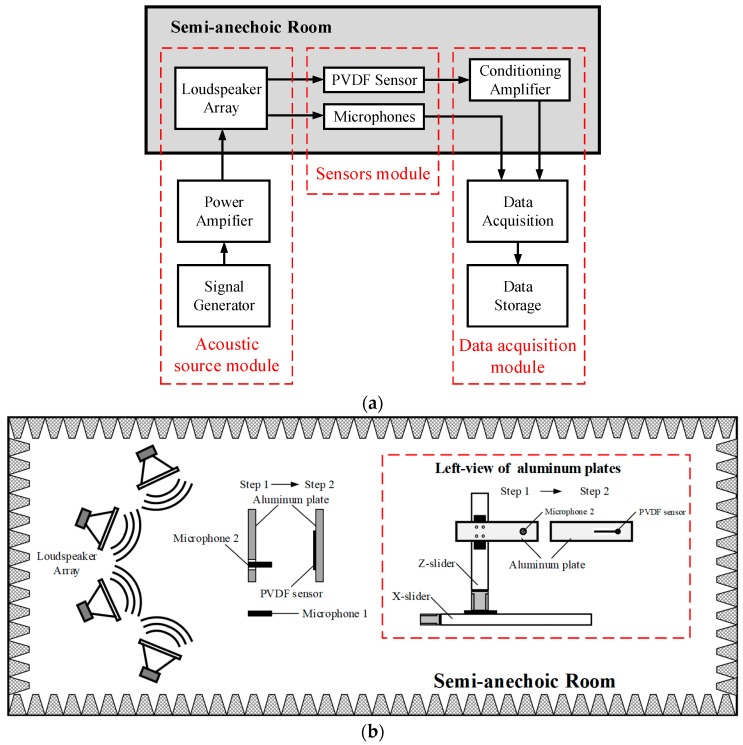
Schematic diagram of the calibration apparatus: (**a**) block diagram of the calibration system. (**b**) detailed schematic diagram in semi-anechoic room.

The calibration apparatus consists of an acoustic source module, sensors module, and data acquisition module, as Figure 4a shows. The experiment was performed in a semi-anechoic room, and the detail schematic diagram in the room is shown in Figure 4b. Signal generated by a four-channel arbitrary waveform card was amplified by the power amplifier and then sent into the loudspeaker to produce sound pressure load on the PVDF sensor and the microphones. By phase correction of the four loudspeakers, sound pressure could be superposed at a given position with up to 20 kHz frequency and 4.0 kPa amplitude. Sensors module contained the PVDF sensor waiting for calibration and two microphones. Microphone 1 was used to confirm the consistency of the sound field between the same test conditions. Microphone 2 was used to measure the actual pressure loading on the sensor. The characteristic parameters of the sensor could be obtained by comparing the output of the sensor and Microphone 2. Electrical charges generated by the sensor were converted into voltage signals by a conditioning amplifier.

### 3.2. Calibration Results

**Figure 5:**
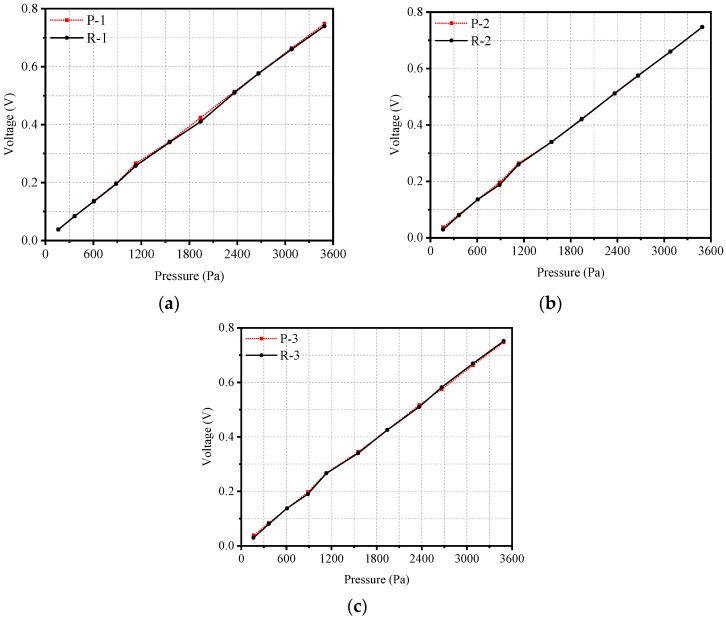
Calibration results for three cycles (“P” represents positive stroke; “R” represents reverse stroke, the same below): (**a**) pressure cycle 1, (**b**) pressure cycle 2, (**c**) pressure cycle 3.

The calibration was performed under the industry standard of piezoelectric pressure sensor of Standards of Machinery Industry of the People’s Republic of China (JB/7482-2008). In this calibration, 11 values from 160–3500 Pa with three forward and reverse loadings were performed, as Figure 5 shows. The horizontal and vertical axes represent the output amplitude of Microphone 2 and PVDF sensor, respectively. Data was sampled at 20 kHz for 20 k samples, which gave a frequency resolution of 1 Hz (the same as below). The output of the PVDF sensor presented a high signal-to-noise ratio and a good consistency with the input dynamic sound pressure.

Sensitivity is defined as the ratio of output change to input change in steady state. The average sensitivity of the sensor was 1.987 pC/N. The *d*_33_ of the PVDF material (see in Table 1) was about 21 pC/N, this difference may be due to the existence of protective layer, shieling layer and the electrodes, as well as the fabrication process. Hence, calibration was necessary and important for PVDF sensor and it was inaccurate to take the *d*_33_ as the sensor sensitivity.

**Figure 6:**
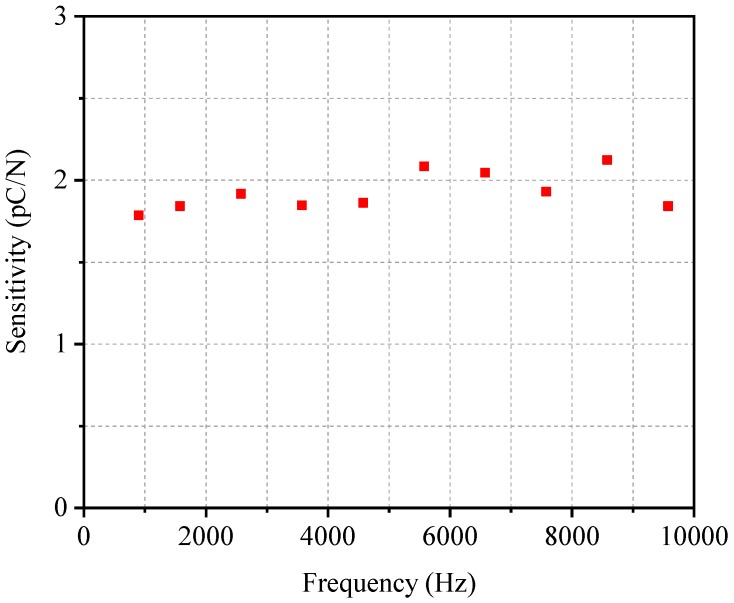
Calibration result for frequency response.

According to the results in Figure 5, the values of the linearity, hysteresis error, and standard deviation are 0.73%, 1.04%, 0.44%, respectively. They all below 2.5% required by the industry standards. Ten frequencies within 10 kHz were calibrated to obtain the frequency response. Figure 6 shows the calibration result for frequency response. It can be seen from Figure 6 that the sensitivity of the sensor changed little within the frequency range of 10 kHz. The standard deviation value is 9.73%, which is below 30% required by the dynamic pressure measurement. The calibration indicates that the sensor can not only meet the industry standards, but also fulfill the requirements of dynamic pressure measurement.

**Figure 7:**
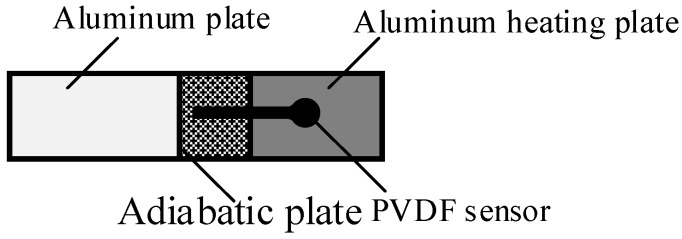
Schematic diagram of the aluminum heating plate.

In axial flow compressor, temperature was gradually increasing with speed, so it was necessary to research the influence of temperature on the PVDF sensor. In this calibration, a heating plate with an inner thermometer was adopted to provide a constant temperature environment, as Figure 7 shows. The aluminum plate was used for fixing. In order to minimize the interference of the external environment, an adiabatic plate was used to connect the aluminum heating plate and the aluminum plate.

**Figure 8:**
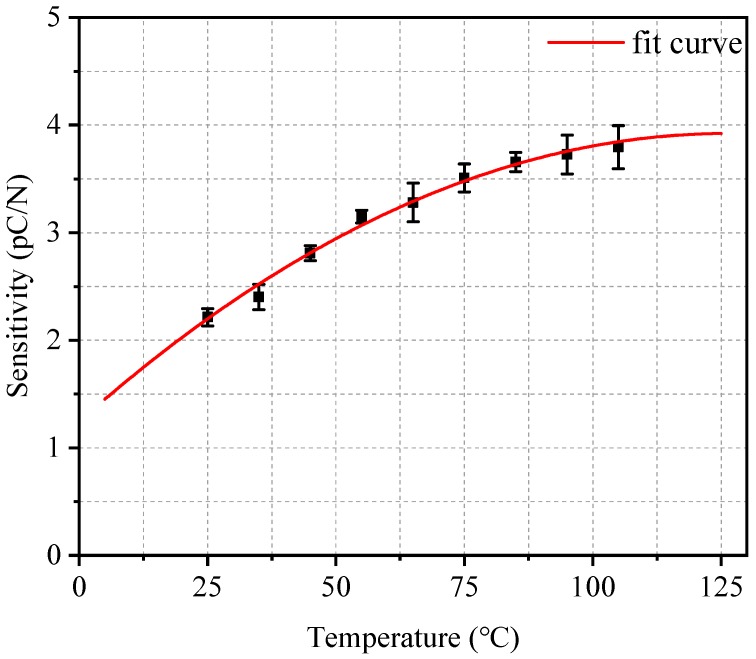
The relation between sensitivity and temperature.

The temperature level increased from 25 to 105 °C in 10 °C steps. The PVDF sensor output increased with the temperature and presented a high signal-to-noise ratio at different temperatures. Figure 8 shows the relation between the sensitivity and the temperature. Three forward and reverse loadings were performed, the sensitivity values for each temperature are average ones. The hysteresis error value is 5.27%. It can be seen from the figure that the sensor sensitivity increased with the temperature. From 25 to 105 °C, the sensor sensitivity increased as much as 71.35%, hence temperature should be taken into consideration for practical use, especially in a temperature-change environment. In order to quantify this variation, polynomial fit was adopted, and the fitting result was:

(1) $$S=-2.1\times 10^{-4}t^{2}+0.04783t+1.08815$$

where *t* (°C) is the temperature.

## 4. Experimental Setup

**Figure 9:**
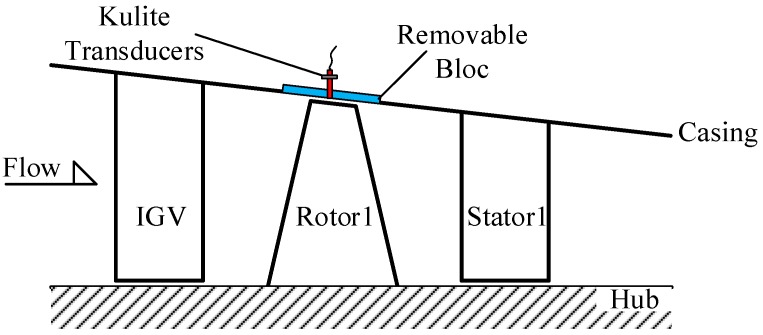
Schematic of test compressor.

**Figure 10:**
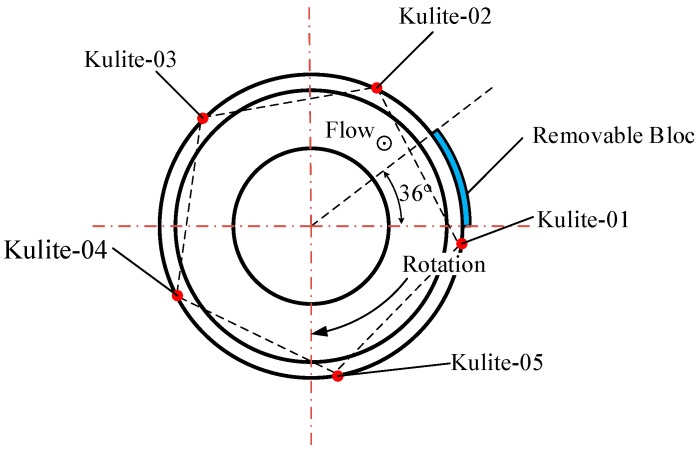
Relative position in circumference of the PVDF sensor array and Kulite transducers.

The test rig is a high-speed 3.5-stage axial flow compressor. Figure 9 shows a schematic of the test compressor. The number of blades of the guide vane, first rotor, and first stator were 42, 38 and 82, respectively. The PVDF sensor array was glued to a removable bloc and then the bloc was fixed on the casing in the range of the region of the first rotor blade, as Figure 10 shows. For validation, 5 high-response pressure transducers were equidistantly spaced around the casing wall over the first blade (represented by Kulite-01–05). The pressure range of the Kulite transducers was 1.7 bar (absolute pressure), which was sufficient for the pressure measurement of the compressor. Their relative positions in axial and circumferential direction are shown in Figure 9 and Figure 10, respectively.

**Figure 11:**
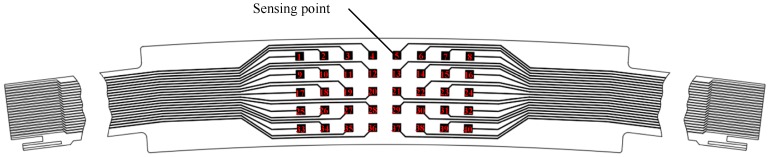
Sketch diagram of the PVDF sensor array.

**Figure 12:**
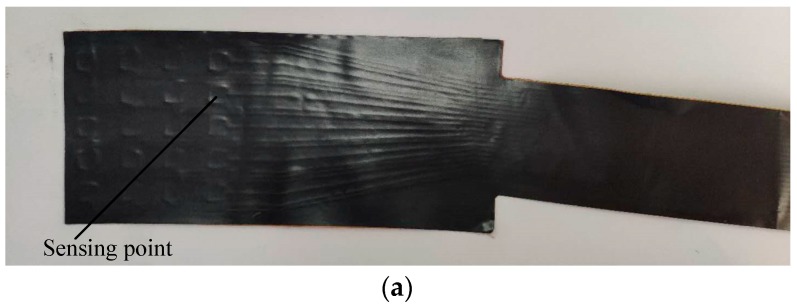

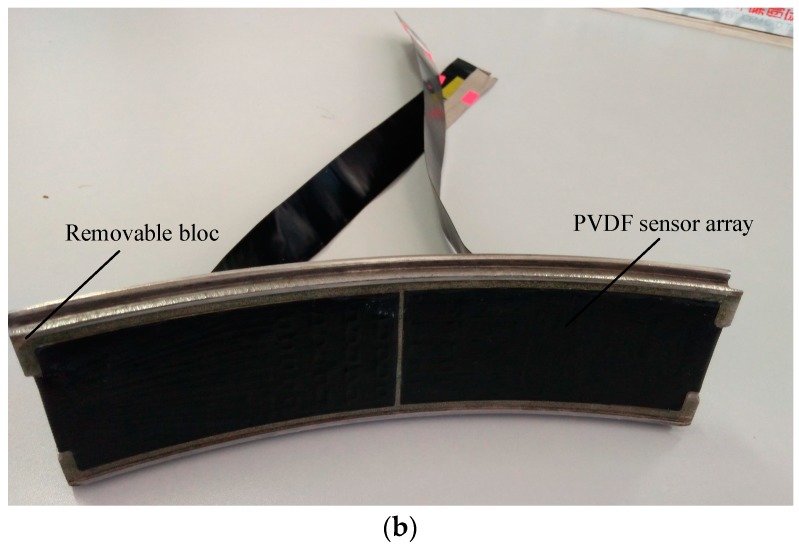
Photograph of the PVDF sensor array: (**a**) sensor photography, (**b**) installation photography.

**Figure 13:**
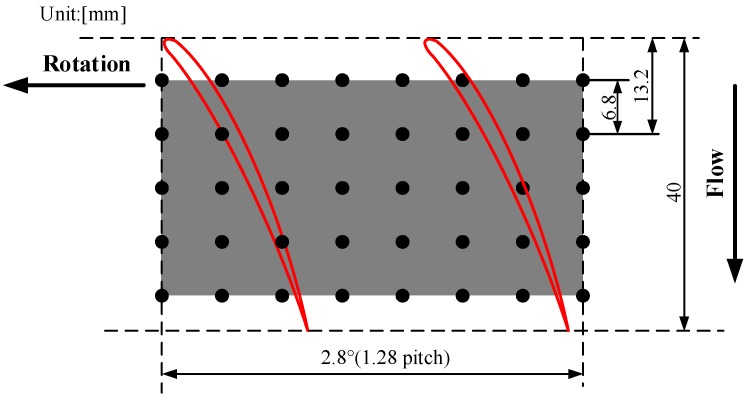
Relative position of the sensing points and the rotor blade.

The PVDF sensor array was designed to be curved according to the spatial structure of the casing, the sketch diagram is shown in Figure 11. The number in the box refers to the position of the channel corresponding to each sensing point. The PVDF sensor array comprises five rows at 6.8-mm intervals in the axial direction and eight columns at 0.4° intervals in the circumferential direction, where the spacing of eight columns is nearly equivalent to the 1.28 pitch of the rotor blade. When the sensor was glued on the casing wall, the axial and circumferential resolution of the sensor was 6.8 mm and 1.73°, respectively. The sensor could only measure the average pressure of each sensing point since the charges conducted from each sensing point were concerned with resultant force on the whole measurement area. Therefore, the sensing point should be small enough to guarantee the measuring accuracy on a spatial point, while large enough to obtain an obvious output charge signal. Taking all factors into consideration, each sensing point was set to be a 3 mm × 3 mm square, thus the area of each point was 9 mm^2^, as Figure 11 shows. Figure 12a shows the photograph of the PVDF sensor array and (b) shows the photograph when the sensor was already glued on the removable bloc. Figure 13 shows the relative position of the sensing points and the rotor blade.

The PVDF sensor array was glued to the casing wall over the first rotor blade tip. Then the sensor was connected to an external conditioning amplifier to convert the charge signal into a voltage signal. Low noise cable should be applied in this connection since the charge signal is susceptible to outside interference such as cable vibration in transmission. All 40 channel signals were acquired simultaneously by the data acquisition system, and then stored by the computer. Since the temperature was rising in the experiment, a temperature sensor was installed near the PVDF sensor array in order to modify the influence of the temperature. The sensitivity of the sensor was corrected in real time in the acquisition software through the sensitivity-temperature relation equation (Equation (1)).

## 5. Experimental Results

### 5.1. Single Sensing Point Results and Comparison

**Figure 14:**
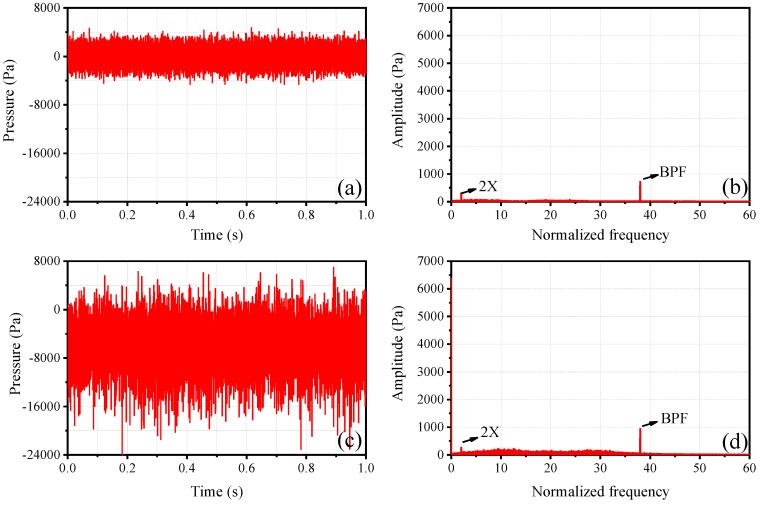
(**a**,**b**) Time domain and frequency domain of PVDF-02, (**c**,**d**) time domain and frequency domain of Kulite-01.

In order to validate the effectiveness of the PVDF sensor array, single sensing point results are compared with Kulite transducers in the time domain and frequency domain. The compressor is in stable operation and the dimensionless rotational speed is about 0.53, which is defined as the ratio of the experimental rotational speed to the designed rotational speed. The sampling frequency (sf) is 50 kHz. Figure 14a,b show the time domain and frequency domain of PVDF-02. The frequency of the abscissa axis has been normalized by the rotating frequency (the same below). The time length is 1 s. It can be seen from Figure 14b that in stable operation, the blade passing frequency (BPF) is dominated in the frequency spectrum, which is due to the rotor blades sweeping the sensor alternately. Figure 14c,d show the time domain and frequency domain of Kulite-01, the position of which is close to PVDF-02. The PVDF sensor is a piezoelectric sensor that can only measure dynamic pressure signals, while the Kulite transducers are piezoresistive, which means that both dynamic and static pressure signals can be measured by them. Due to a negative pressure at this time and location, the fluctuation of Kulite-01(Figure 14c) shifts to the minus side compared with PVDF-02 (Figure 14a). Comparing Figure 14b,d, the 2X and BPF are both contained in the frequency spectrums of PVDF-02 and Kulite-01, and the BPF is dominant. At the same time, the amplitudes of their main frequencies are basically the same. The results of other sensing points have the same conclusions, and this indicates that the self-developed PVDF sensor array is effective.

**Figure 15:**
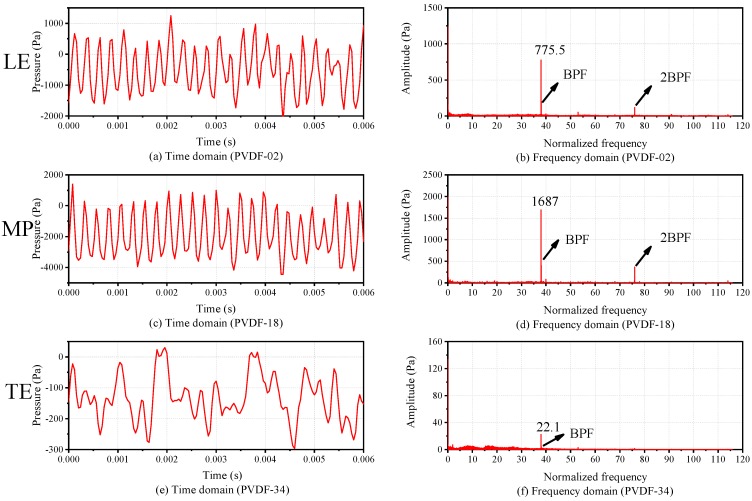
Time domain and frequency domain comparison in axial direction.

Figure 15 shows the time domain and frequency domain comparison in the axial direction. Comparing the time domain of three sensing points PVDF-02, 18, 34 (the positions of them can be referred to the numbers in the box shown in Figure 11) along the axial direction, it can be seen that the waveforms of the three sensing points have certain periodicity. Among them, the waveform of PVDF-18, which is located in the middle part (MP) over the blade tip, has the best periodicity. By contrast, the waveform of PVDF-34, which is located in the trailing edge (TE) over the blade tip, has the worst periodicity. This is owing to the fact that the position near the TE of the blade tip is affected by the secondary flow, such as the wake flow and rotor-to-stator interference. When the flow with a large amount of pulsation in the MP of the blade tip reaches this position, the pulsation amplitude decreases, and the periodicity deteriorates. Comparing the frequency domain of them, the amplitude of the BPF shows an upward trend from the leading edge (LE) to the MP, and then a downward trend from the MP to the TE. In the MP over the blade tip, the flow field pressure changes most in the circumferential direction, since the pressure difference is equal to the pressure difference between the pressure side and the suction side of the blade. Therefore, the pressure pulsation in the MP over the blade tip is the largest compared with other axial positions.

### 5.2. Casing Wall Dynamic Pressure Field (Stable State)

**Figure 16:**
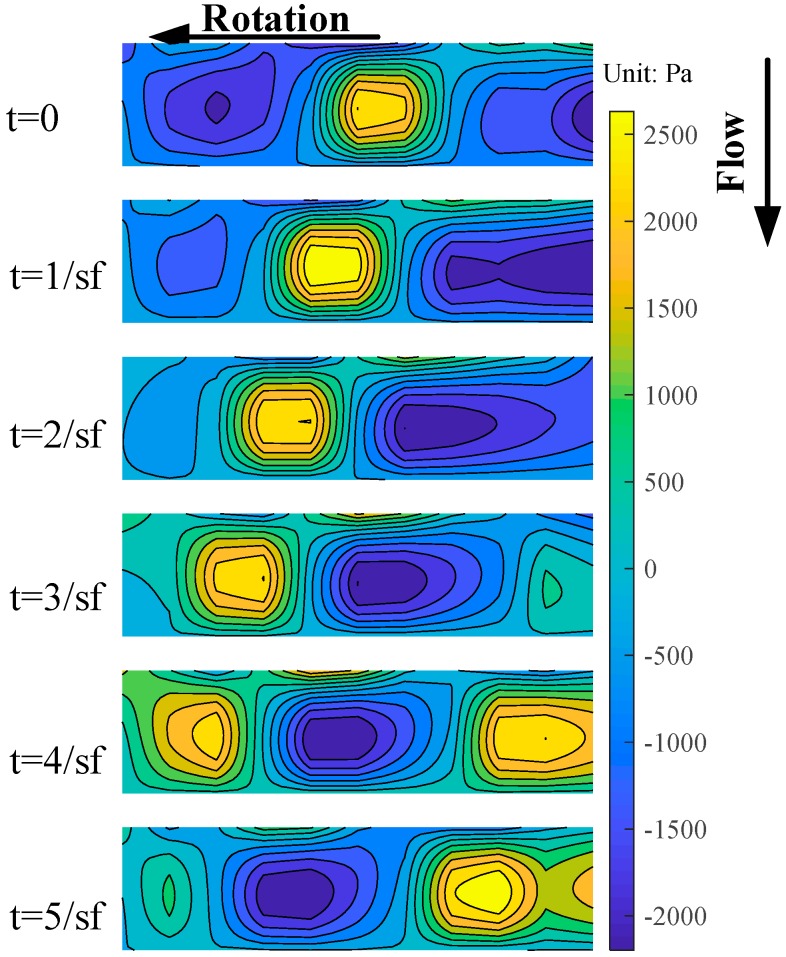
Casing wall dynamic pressure field.

Figure 16 shows the casing wall dynamic pressure field drawn by the spatial interpolation [14], and the compressor is under stable operation with 0.53 designed speed. The six subgraphs from top to bottom in the figure represent the pressure field of six consecutive sampling moments. It can be seen from the figure that the high-pressure spot and the low-pressure spot are alternately arranged in the dynamic pressure field. They are formed by the pressure side and suction side of the blade, respectively. Due to the blades sweeping alternately above the PVDF sensor array, they propagate in the direction of the rotation with time. This is consistent with the pressure distribution measured by common pressure transducers in the existing literature.

### 5.3. Casing Wall Dynamic Pressure Field (NSV State)

**Figure 17:**
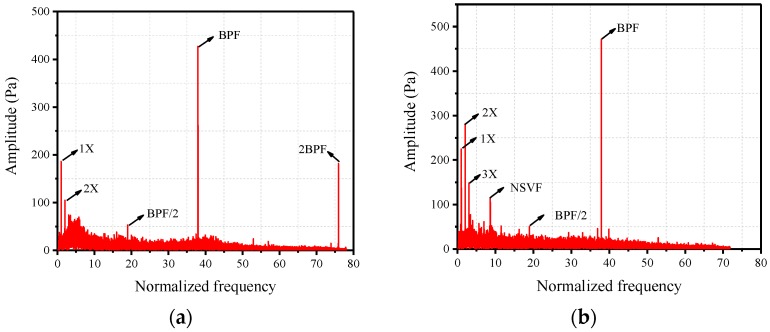
Frequency domain comparison of stable state and non-synchronous vibration (NSV) state (PVDF-01): (**a**) stable state (**b**) NSV state.

**Figure 18:**
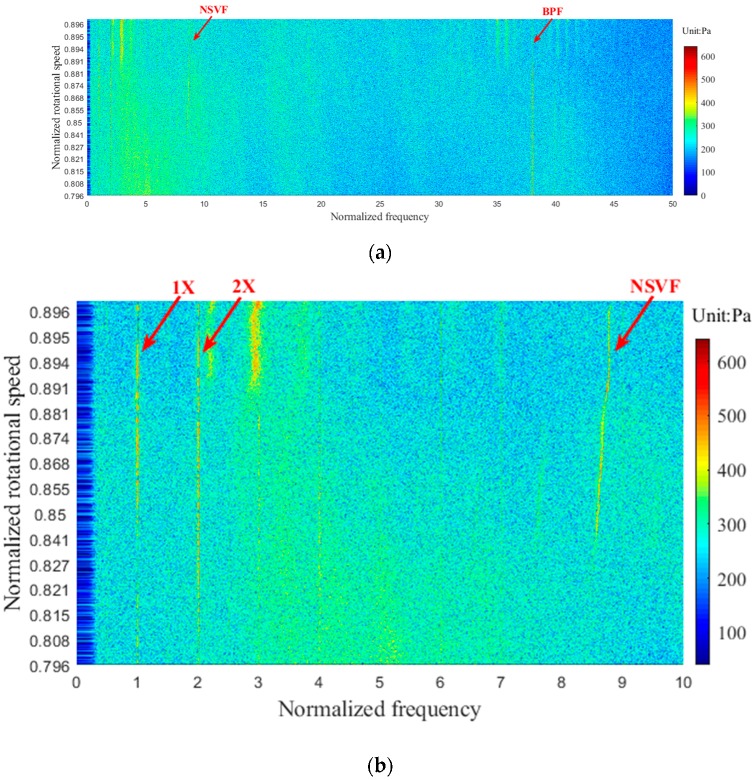
Waterfall plot of the NSV state (PVDF-01): (**a**) normalized frequency range 0–50, (**b**) normalized frequency range 0–10.

In recent years, blade fatigue and cracks due to the NSV (also known as the acoustic resonance) of the rotor has been of increasing concern, and a number of experimental studies and numerical simulations have been performed to research the mechanism of NSV. Although various hypotheses have been proposed to try to explain this phenomenon, no conclusion has been reached so far. Nowadays, more and more scholars begin to study NSV from the perspective of tip clearance flow [7,8,9]. In this experiment, the PVDF sensor array, which is glued to the casing wall of the first rotor blade tip, has also captured this phenomenon. When the first rotor blade was subjected to high amplitude vibration, the NSV has been captured by all sensing points of the sensor. Figure 17 shows the frequency spectrums of two states (stable state and NSV state) of PVDF-01. The data has been processed by a high-pass filter to remove some high-amplitude low-frequency noises. The NSV frequency is a non-integer multiple of the rotating frequency (1X), about 8.76 times to rotating frequency. Figure 18 shows the waterfall plots of PVDF-01 when the rotational speed is from 0.796–0.901 designed speed. Figure 18b is the result of limiting the normalized frequency range of Figure 18a to 0–10. It can be seen from Figure 18b that as the speed rises, the NSV frequency starts to appear at 0.841 designed speed, reaches its maximum at 0.891 designed speed and disappears at 0.896 designed speed. In this process, the BPF is a predominant component at the beginning, while decreasing after the emergence of the NSV frequency, and the amplitude of NSV frequency begins to increase and even exceed the BPF. Moreover, the frequency of NSV gradually increases with the rotational speed. These phenomena are consistent with the results observed in the literature [30,31].

**Figure 19:**
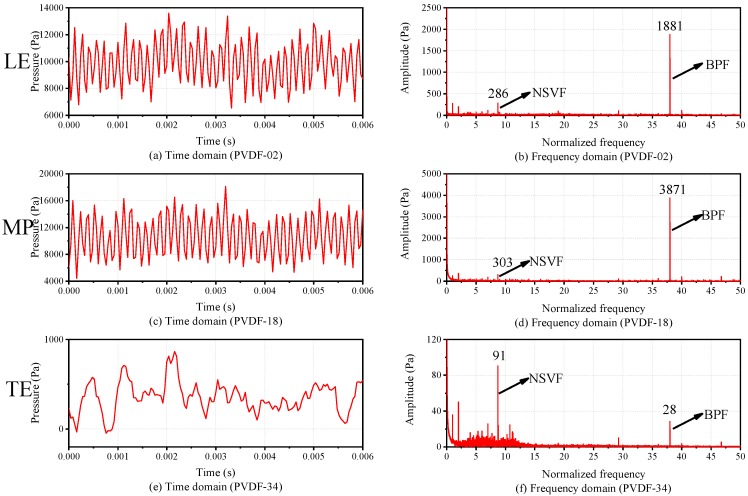
Time domain and frequency domain comparison in the axial direction (NSV state).

Figure 19 shows the time domain and frequency domain comparison in the axial direction (NSV state). Comparing with stable state (Figure 15), the trend of BPF amplitude is similar, both of them show an upward trend from the LE to the MP, and a downward trend from the MP to the TE. But in NSV state, the NSVF component appears in the frequency spectrum in all sensing points, and the amplitude trend of NSVF is consistent with BPF, both of them reach maximum value in the MP of the blade tip. Moreover, BPF always dominates in the frequency spectrums in the LE and MP, while NSVF dominates in the frequency spectrum in the TE, and it exceeds the BPF.

**Figure 20:**
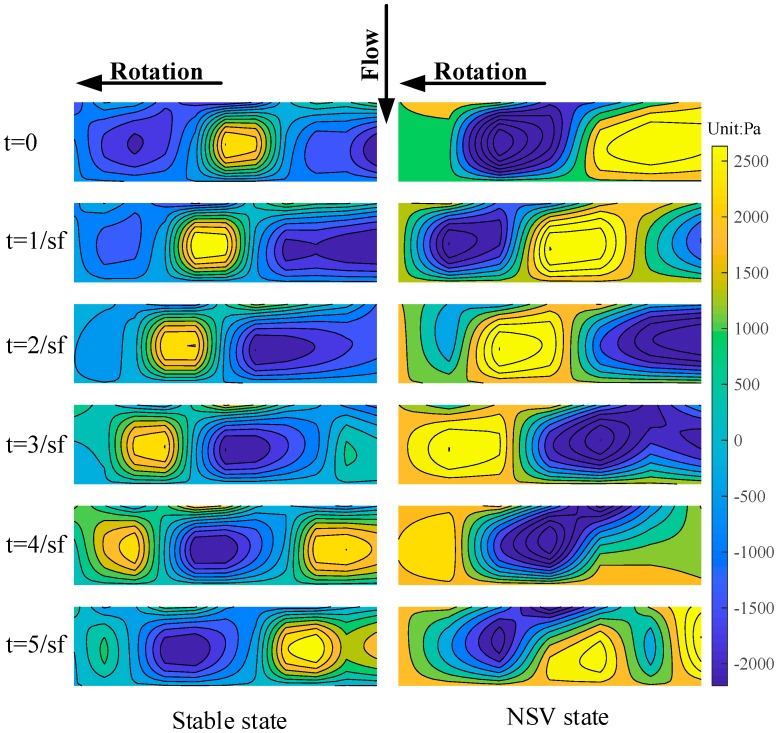
Dynamic pressure field comparison of stable state and NSV state.

Figure 20 shows the dynamic pressure field comparison of stable state and NSV state, and both of them are drawn by spatial interpolation. Comparing the dynamic pressure fields at the six moments of the two states, the vortex size, as well as the flow field in different blade intervals, are basically the same, and the vortex shape is regular in the stable state. In the case of NSV, due to the presence of NSVF, the vortex size changes with time, and there is disturbance in the flow field. At some moments, the high-pressure spot occupies the entire area from the TE to the LE over the blade tip.

In summary, in view of the advantages of the aforementioned PVDF sensor array, it may bring a new breakthrough for the mechanism research of NSV.

## 6. Conclusions

There are two objectives of the present work: the development of a PVDF sensor array for the dynamic pressure field measurement over the blade tip in an axial flow compressor and the evaluation of its performance by a practical application. Dynamic calibration results indicate that the sensor presents a high signal-to-noise ratio and a good consistence with the reference microphone. Noteworthy, temperature has a great influence on the sensitivity, hence necessary consideration is needed. The calibration gives credence to the relevance and reliability of this sensor for application in dynamic pressure field measurement. A practical application of the sensor is performed in a compressor. Measurement result of the sensor also maintains good consistence with common high-response pressure transducers. Dynamic pressure field of the blade tip is obtained by means of spatial interpolation. The NSV is also captured by the sensor and the characteristics of the dynamic pressure field when the compressor is under stable state and NSV state are revealed. In view of the advantages over common pressure transducers, the PVDF sensor array may provide a high-resolution, inexpensive and undamaged technology for dynamic pressure field measurement, especially for pressure measurement of the complex spatial surface or thin-walled structure, such as the blade surface and the thin casing wall of the compressor.

## 7. Patents

Thin film sensor array calibration device and calibration method based on sound pressure loading (CN201610744025).

## Figures and Tables

**Table 1:** Basic parameters of polyvinylidene fluoride (PVDF) material.

Items	Units	Value
Piezoelectric Strain Constant *d*_33_	pC/N	21
Piezoelectric Voltage Constant *g*_33_	V·m/N	0.2
Frequency Response	Hz	10^−2^ to 10^8^
Pressure Range	Gpa	0 to 20
Acoustic Impedance	Pa·s/m^3^	2.5–3 × 10^5^
Yield Strength	N/m^2^	45–55 × 10^6^
Density	kg/m^3^	1.78 × 10^3^
Poisson’s Ratio		0.3
Operating Temperature Range	°C	−40 to 80
Thickness	µm	30

**Table 2:** Comparison between the PVDF sensor and common pressure transducers ^1^.

Sensor	PVDF Sensor	Kulite XCQ-062	Kulite LQ-062	Endevco 8507C-15	Endevco 8515C-50
Picture	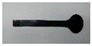	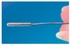	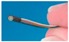	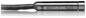	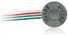
Operational Mode	Gage	Absolute, Sealed Gage	Absolute, Sealed Gage	Gage	Gage
Price (RMB)	500	9600	9500	16,000	12,000
Installation	Glued on the surface	Flush mounted	Glued on the surface	Flush mounted	Surface-mounted
Frequency response (Hz)	tested up to 10 kHz	0–2 × 10^5^	0–2 × 10^5^	0–2.6 × 10^4^	0–6.4 × 10^4^
Diameter (mm)	≥2.3 mm	1.7	1.6	2.3	6.3
Thickness/Length ^2^ (mm)	0.36	9.5	0.76	12.7	0.76
Pressure range (PSI)	tested up to 3.5 kPa	1000	1000	15	50

^1^ The parameters of the Kulite and Endevco transducers in the table are partly from the official website and partly from the market. ^2^ For PVDF sensor, Kulite LQ-062 and Endevco 8515C-50, this parameter indicates thickness. For Kulite XCQ-062 and Endevco 8507C-15, this parameter indicates length.

## Abbreviation

PVDF	polyvinylidene fluoride
PVDF sensor array	PVDF piezoelectric-film sensor array
PVDF-01–40	sensing point 1–40 of the PVDF sensor
Kulite-01–05	Kulite high-response pressure transducer 1–5
1X	rotating frequency
2X	second harmonic frequency
3X	third harmonic frequency
BPF	blade passing frequency
NSV	non-synchronous vibration
NSVF	non-synchronous vibration frequency
sf	sampling frequency
LE	leading edge
MP	middle part
TE	trailing edge
